# Mr. Harrold, on Crusta Lactea

**Published:** 1806-01

**Authors:** E. Harrold

**Affiliations:** Cheshunt, Herts


### *To the Editors of the Medical and Physical Journal.*

Gentlemen,

I Do not recollect that any of your Correspondents have written on the subject of Crusta Lactea. This sometimes proves a very severe and troublesome disease; in some families attacking every child at the age of a few weeks, and continuing with more or less violence till the period of teething is over. I have attended several children in a family in this village, who have had it most severely. The eruption was not confined to the head and face, but extended to the arms and different parts of the trunk. The irritation was so great, that it frequently required for many nights and days together, the constant care of a servant, to prevent my little patients from scratching themselves, which they frequently did, till they bled profusely. The discharge from different parts of the skin, made their caps and linen as stiff as horn, and to adhere so firmly as not to be easily removed without also taking away the cuticle to which they adhered. It has been sometimes found necessary, not only to muffle their hands, but to pin them to the bed-clothes; though I have never seen any permanent marks, and have sometimes thought this superficial bleeding useful. This excessive irritation and want of rest were sometimes attended by loss of appetite, and consequent loss of flesh and strength. The two oldest were very much benefited by taking asses milk, and by the occasional use of bark, natron, &c. as ordered for them, by the justly celebrated Doctor Willan. They were inoculated with small-pox during the active state of the disease; both had it mildly, but it appeared to make no alteration in the state of the original complaint. The second, a girl, now about 6 years of age, has not been, till lately, entirely free from some of the effects of the disorder about her mouth. The third child, a boy, when 6 weeks old, was attacked in the same manner as the two elder children. The complaint soon extended itself over a variety of parts of the body, the bends of the arms, &c. and threatened to be as severe as it proved with the two others; but at this time, by the recommendation of Dr. Willan, the child was vaccinated; he had a true vaccine vesicle, and on the ninth day, when the areola began to extend itself, the general irritation of the skin vanished. The child was afterwards not without appearances of the disease, but it was unaccompanied by any degree of irritation, and was very soon removed entirely.

The first three children having been attacked so decidedly, it was reasonable to expect that the fourth would not escape; but a preventive was mentioned to me by its anxious mother, and said to have been the practice of Dr. Orme in such cases. As I have never had the pleasure of the Doctor's acquaintance, I cannot tell how far this may be true; but perhaps the medical world may be favoured with some farther intelligence through the medium of the Medical and Physical Journal, and for which I, for one, shall be very thankful. On the day after the *fourth* child was born, a small blister was applied inter scapul. When it had drawn properly it was suffered to heal, and then a second was applied, which was kept open with cerat sabinæ till the child was near three weeks old. He was evidently weakened by it, and lost flesh, but he has never had the slightest appearance of eruption of any kind, though he is now almost 2 ½ years old, and is a fine healthy child.

A *fifth* child, a boy, was born on the 7th of last September, and on the following day a blister was applied, and managed exactly in the same way as with the fourth; this child, however, was certainly more robust than the former one, and did not suffer so much in his general health by the remedy; but, whether this circumstance is, or is not material I cannot tell, but at the end of six weeks, there certainly were some appearances very much like a commencement of the disease; and that it might not have time to establish itself, another blister was applied and kept open, and on the 22d of October he was vaccinated. After these remedies, every vestige of the disease disappeared, and there has hitherto been no return. To those practitioners who have frequently seen this complaint in its most troublesome form, these remarks may be of some value; and if any Gentleman's experience sanctions this mode of treatment, or can point out a better, he will discharge an important duty, by communicating it to the world.

I am, &c.

*Cheshunt, Herts, Nov. 25, 1805.* E. HARROLD

